# Effect of cryopreservation and lyophilization on viability and growth of strict anaerobic human gut microbes

**DOI:** 10.1111/1751-7915.13265

**Published:** 2018-04-17

**Authors:** Lea Bircher, Annelies Geirnaert, Frederik Hammes, Christophe Lacroix, Clarissa Schwab

**Affiliations:** ^1^ Laboratory of Food Biotechnology Institute of Food, Nutrition and Health ETH Zürich Schmelzbergstrasse 7 8092 Zürich Switzerland; ^2^ EAWAG Überlandstrasse 133 8600 Dübendorf Switzerland

## Abstract

Strict anaerobic gut microbes have been suggested as ‘next‐generation probiotics’ for treating several intestinal disorders. The development of preservation techniques is of major importance for therapeutic application. This study investigated cryopreservation (−80°C) and lyophilization survival and storage stability (4°C for 3 months) of the strict anaerobic gut microbes *Bacteroides thetaiotaomicron*,* Faecalibacterium prausnitzii*,* Roseburia intestinalis*,* Anaerostipes caccae*,* Eubacterium hallii* and *Blautia obeum*. To improve preservation survival, protectants sucrose and inulin (both 5% w/v) were added for lyophilization and were also combined with glycerol (15% v/v) for cryopreservation. Bacterial fitness, evaluated by maximum growth rate and lag phase, viability and membrane integrity were determined using a standardized growth assay and by flow cytometry as markers for preservation resistance. Lyophilization was more detrimental to viability and fitness than cryopreservation, but led to better storage stability. Adding sucrose and inulin enhanced viability and the proportion of intact cells during lyophilization of all strains. Viability of protectant‐free *B*. *thetaiotaomicron*,* A. caccae* and *F. prausnitzii* was above 50% after cryopreservation and storage and increased to above 80% if protectants were present. The addition of glycerol, sucrose and inulin strongly enhanced the viability of *B. obeum*,* E. hallii* and *R. intestinalis* from 0.03–2% in protectant‐free cultures to 11–37%. This is the first study that quantitatively compared the effect of cryopreservation and lyophilization and the addition of selected protectants on viability and fitness of six strict anaerobic gut microbes. Our results suggest that efficiency of protectants is process‐ and species‐specific.

## Introduction

According to the WHO/FAO, probiotics are defined as ‘live microorganisms, that when administered in adequate amounts confer a health benefit on the host’. This definition implies viability as an important characteristic of probiotics for efficiency. Processing and preservation procedures that guarantee a high yield of viable cells are therefore essential for probiotic application. Recently, interest in probiotic research expanded from the classical probiotic *Lactobacillus* and *Bifidobacterium* species to a more targeted manipulation of the host gut microbiota with ‘personalized probiotic therapies’ using functional important strict anaerobic gut microbes. The administration of specific bacterial strains could address distinct differences in colonic microbiota profiles associated with intestinal diseases (Vieira *et al*., 2016). Strict anaerobic butyrate‐producing bacteria have been proposed as ‘next‐generation probiotics’ in the treatment of intestinal disorders (Van Immerseel *et al*., 2010). Butyrate, produced during bacterial fermentation, is an important short‐chain fatty acid (SCFA) providing several benefits to the host (Tan *et al*., 2014). Clostridia cluster IV and XIVa, including the highly abundant butyrate‐producing *Faecalibacterium prausnitzii* and *Eubacterium rectale/Roseburia* spp., harbour promising candidates for future probiotics (Louis and Flint, 2009; Hsiao *et al*., 2014; Miquel *et al*., 2015; Udayappan *et al*., 2016; Tamanai‐Shacoori *et al*., 2017). The selection of ‘next‐generation probiotics’, however, is not limited to butyrate producers. Propionate producers, such as *Bacteroides*, can beneficially effect the host by interacting with the immune system and by maintaining host–microbiota homoeostasis and might therefore contain species for future therapeutic administration (Wrzosek *et al*., 2013; El Hage *et al*., 2017).

The two main long‐term preservation methods for microbes are cryopreservation and lyophilization (Prakash *et al*., 2013). Both techniques are well established for many aerobes or facultative anaerobes (Hubalek, 2003). However, data on preservation of strict anaerobic gut microbes are limited (Staab and Ely, 1987; Khan *et al*., 2014). Current preservation procedures were mainly designed for culture collections when survival of only a minor proportion of cells is required (Malik, 1992). To improve survival of preserved cells, protective agents are commonly added to minimize freezing and drying injuries. In cryopreservation, glycerol is one of the most commonly used penetrating protectant; it is non‐toxic even at high concentrations (Meryman, 2007). Intracellular glycerol can stabilize cells during slow freezing by minimizing or delaying osmotic derived shrinkage off the cells to a lower temperature (Fowler and Toner, 2005). It has been suggested that glycerol can prevent damage due to increased osmotic pressure, as the presence of glycerol can reduce the excessive increase in salt concentration in fractions of unfrozen water during freezing (Lovelock, 1953). In contrast, in lyophilization glycerol is less suitable as protectants, because it can lead to an insufficiently dried and sticky product at high concentrations (Abadias *et al*., 2001). Sugars are another classical group of protectants used in cryopreservation and lyophilization. Disaccharides, such as maltose, sucrose and trehalose, are able to induce shrinkage of the cells by osmosis‐derived dehydration before freezing thereby reducing intracellular ice formation (Fowler and Toner, 2005). Sucrose has been frequently used for cryopreservation of microorganism (Hubalek, 2003) and improved tolerance to drying by protecting proteins from denaturation in the absence of water (Leslie *et al*., 1995). Inulin‐type fructans are non‐penetrating, water‐soluble protective agents that are applied in lyophilization (Hubalek, 2003). The protective action is exerted extracellularly by direct interaction and stabilization of membrane lipids under dry and cold conditions (Demel *et al*., 1998; Vereyken *et al*., 2003; Schwab *et al*., 2007). Fructans can serve as bulking agent and protective matrix during lyophilization (Khan *et al*., 2014). Combining compounds with different protective mechanisms can result in greater protection of microorganisms during freezing and drying than single‐component application, due to additive or synergic protective effects (Hubalek, 2003). We recently showed, that a combination of glycerol (15% v/v) and inulin (5% v/w) maintained viability and activity of the strict anaerobic, butyrate‐producing microbes, *F. prausnitzii*,* Roseburia* sp./*E. rectale* group and *Eubacterium hallii* in complex ‘artificial’ gut microbial communities during 3 months storage at −80°C (Bircher *et al*., 2017).

In this work, we investigated preservation of strict anaerobic gut microbes after freezing at −80°C and after lyophilization, and subsequent storage at 4°C for 3 months. Six strains were selected for preservation trials: *Roseburia intestinalis*,* F. prausnitzii*,* E. hallii, Anaerostipes caccae, Blautia obeum* and *Bacteroides thetaiotaomicron* are highly abundant representatives of human gut microbial butyrate and propionate producers. Bacterial fitness, evaluated by maximum growth rate and lag phase, and viability were tested during processing under strict anaerobic conditions and storage using different buffers containing non‐toxic protectants glycerol, sucrose and inulin to improve freezing and freeze‐drying resistance.

## Results

### Impact of protectants on fresh cultures

**Figure 1:**
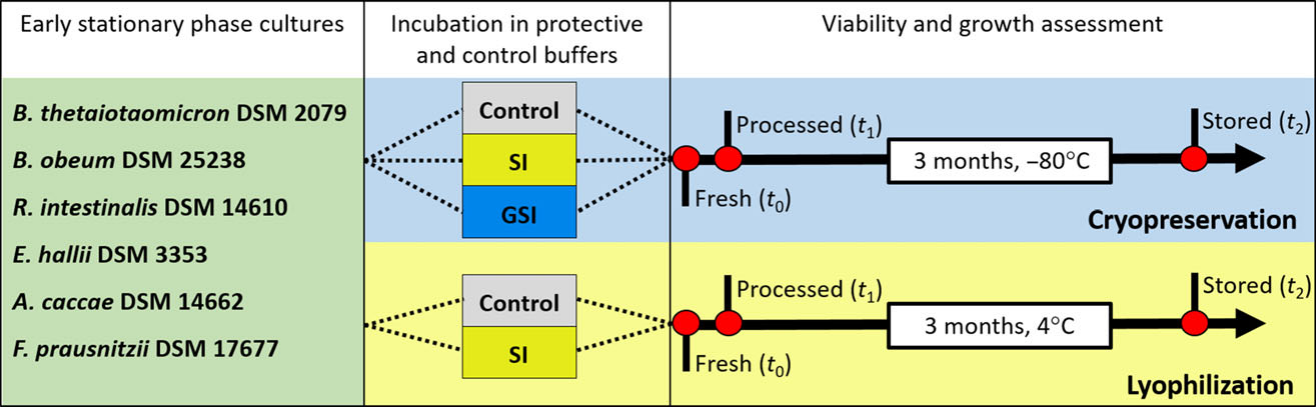
Set‐up of preservation experiments. Early stationary phase cultures were incubated for 30 min in buffers containing sucrose and inulin (SI), sucrose, inulin and glycerol (GSI) and in control buffer lacking protectants (control) before processing for preservation. Viability and growth were assessed at three different time points indicated by red dots. The first assessment was performed with fresh cultures after incubation in the protective and control buffers (*t*_0_). The second assessment was conducted with the processed cultures immediately after freezing, respectively, lyophilization (*t*_1_) and the third assessment with cryopreserved and lyophilized samples stored for 3 months at −80°C or 4°C, respectively (*t*_2_).

The effect of the protectants inulin and sucrose alone (SI, bot 5% w/v) or in combination with glycerol (GSI, 15% v/v) on viability (MPNs, percentage of intact cells) and fitness (μ_max_ and *t*_lag_) of fresh *B. thetaiotaomicron*,* B. obeum*,* R. intestinalis*,* E. hallii*,* F. prausnitzii* and *A. caccae* cultures (*t*_0_) was evaluated after 30 min anaerobic incubation in the corresponding protective medium, and compared to a control lacking protectants (Fig. 1).

For all tested strains, SI‐treated cultures did not differ from that of control cultures (Tables 1 and 2), except *A. caccae* with higher percentage of intact cells after incubation in SI than in the control (83 ± 6% and 63 ± 8%, respectively). In contrast, significant differences in viability and fitness of fresh cultures were observed when SI was combined with glycerol (GSI) for some strains. The MPN for *R. intestinalis* cultures was approximately 10‐fold lower with GSI than in the control (7.9 ± 0.2 and 9.0 ± 0.3 log cells ml^−1^, respectively), the percentage of intact cells was reduced (57 ± 5% and 104 ± 6%, respectively), and *t*_lag_ was significantly increased (3.3 ± 0.1 and 1.3 ± 0.1 h, respectively). Similarly, GSI‐treated *F. prausnitzii* exhibited a three times longer *t*_lag_ (3.6 ± 0.1 and 1.3 ± 0.1 h, respectively), a lower fraction of intact cells (65 ± 2% and 83 ± 7%, respectively) and a significantly reduced MPN (7.4 ± 0.3 and 8.0 ± 0.2 log cells ml^−1^, respectively) compared to the control. For *B. thetaiotaomicron* treated with GIS, *t*_lag_ was significantly increased (1.3 ± 0.6 and 0.7 ± 0.1 h, respectively) and the percentage of intact cells was reduced (60 ± 5% and 87 ± 11%, respectively) compared to the control, but MPN was not different. GIS treatment also decreased μ_max_ of *E. hallii* compared to the control (0.23 ± 0.02 and 0.36 ± 0.02 OD unit h^−1^, respectively).

**Table 1:** Impact of protectants and cryopreservation on cell viability of fresh (*t*_0_), processed (*t*_1_) and stored (*t*_2_) bacteria. Log viable cell counts ml^−1^ in the fresh culture, after freezing in liquid nitrogen and cryopreservation for 3 months at −80°C, were assessed with the most probable number method (MPN). Recovery rate of viable cells (in %) was calculated relative to the average viable cell counts in the fresh control (vs. control *t*_0_) and fresh treatment culture (vs. *t*_0_)

Organism	Culture condition	Control		SI		GSI	
		MPN (ml^−1^)	Recovery vs. t_0_	MPN (ml^−1^)	Recover vs. control *t*_0_/*t*_0_	MPN (ml^−1^)	Recovery vs. control *t*_0_/*t*_0_
*B. thetaiotaomicron*	Fresh (*t*_0_)	9.3 ± 0.2		9.4 ± 0.2	135	8.9 ± 0.3	45
	Processed (*t*_1_)	9.2 ± 0.3	85	9.3 ± 0.2	114/84	8.8 ± 0.6	31/70
	Stored (*t*_2_)	9.2 ± 0.2	93	9.3 ± 0.2	100/91	9.0 ± 0.2	60/134
*B. obeum*	Fresh (*t*_0_)	7.9 ± 0.4		7.8 ± 0.4	79	8.1 ± 0.2	139
	Processed (*t*_1_)	5.7 ± 0.7^b^	1	7.1 ± 0.5^a^	15/19	7.7 ± 0.4^a^	57/41
	Stored (*t*_2_)	4.5 ± 0.6^b^	0.03	6.1 ± 0.4^a^^,^ b	2/2	7.0 ± 0.4^a^	11/8
*R. intestinalis*	Fresh (*t*_0_)	9.0 ± 0.3		8.9 ± 0.4	82	7.9 ± 0.2^a^	9
	Processed (*t*_1_)	8.4 ± 0.4^b^	27	8.4 ± 0.3	27/33	8.3 ± 0.3	23/267
	Stored (*t*_2_)	7.1 ± 0.2^b^	1	7.7 ± 0.3^a^^,^ b	6/7	8.3 ± 0.2^a^	21/247
*E. hallii*	Fresh (*t*_0_)	8.4 ± 0.2		8.4 ± 0.2	100	8.3 ± 0.2	81
	Processed (*t*_1_)	7.6 ± 0.1^b^	16	7.8 ± 0.2^b^	25/25	8.1 ± 0.3^a^	52/64
	Stored (*t*_2_)	6.8 ± 0.6^b^	2	7.1 ± 0.4^b^	6/6	7.9 ± 0.4^a^	37/45
*A. caccae*	Fresh (*t*_0_)	8.8 ± 0.3		8.9 ± 0.2	122	8.6 ± 0.4	63
	Processed (*t*_1_)	8.4 ± 0.2^b^	39	8.9 ± 0.2^a^	104/85	8.7 ± 0.2	69/109
	Stored (*t*_2_)	8.5 ± 0.2	49	8.8 ± 0.3	96/78	8.8 ± 0.3	95/152
*F. prausnitzii*	Fresh (*t*_0_)	8.0 ± 0.2		8.3 ± 0.3	181	7.4 ± 0.3^a^	27
	Processed (*t*_1_)	7.9 ± 0.4	76	7.8 ± 0.3^b^	64/36	7.1 ± 0.3^a^	12/46
	Stored (*t*_2_)	7.9 ± 0.2	80	7.6 ± 0.3^b^	45/25	7.2 ± 0.2^a^	16/60

a Viable cell counts in samples with cryoprotectants are significantly different from the control samples within the same condition (*P* ˂ 0.05).

b Viable cell counts after processing and after storage are significantly different from the viable cell counts of the fresh culture within the same treatment (*P* ˂ 0.05).

**Table 2:** Impact of protectants and cryopreservation on fitness of fresh (*t*_0_), processed (*t*_1_) and stored (*t*_2_) bacteria. Lag phase (*t*_lag_) and maximum growth rate (µ_max_) of gut microbes after freezing in liquid nitrogen and cryopreservation for 3 months at −80°C were calculated from optical density growth curves based on Baranyi's equation

Organism	Culture condition	Control		SI		GSI	
		*t*_lag_ (h)	µ_max_ (OD*h^−1^)	*t*_lag_ (h)	µ_max_ (OD*h^−1^)	*t*_lag_ (h)	µ_max_ (OD*h^−1^)
*B. thetaiotaomicron*	Fresh (*t*_0_)	0.7 ± 0.1	0.23 ± 0.01	0.7 ± 0.1	0.22 ± 0.01^a^	1.3 ± 0.6^a^	0.22 ± 0.01^a^
	Processed (*t*_1_)	1.1 ± 0.1^b^	0.22 ± 0.01	0.8 ± 0.1^b^	0.20 ± 0.01^a^	1.6 ± 0.2	0.20 ± 0.01^a^
	Stored (*t*_2_)	0.8 ± 0.3	0.18 ± 0.04^b^	0.5 ± 0.3	0.16 ± 0.04^b^	1.2 ± 0.7	0.17 ± 0.04^b^
*B. obeum*	Fresh (*t*_0_)	6.4 ± 2.2	0.14 ± 0.02	7.0 ± 2.1	0.14 ± 0.02	7.3 ± 1.1	0.13 ± 0.03
	Processed (*t*_1_)	15.1 ± 1.0^b^	0.23 ± 0.01^b^	13.7 ± 2.5^b^	0.17 ± 0.05	13.0 ± 2.5^b^	0.11 ± 0.02^a^
	Stored (*t*_2_)	19.7 ± 0.8^b^	0.26 ± 0.03^b^	13.5 ± 0.7^a^^,^ b	0.21 ± 0.03^a^^,^ b	16.1 ± 2.1^a^^,^ b	0.21 ± 0.03^a^^,^ b
*R. intestinalis*	Fresh (*t*_0_)	1.6 ± 0.1	0.29 ± 0.02	1.6 ± 0.1	0.28 ± 0.01	3.3 ± 0.1^a^	0.26 ± 0.01
	Processed (*t*_1_)	3.2 ± 0.3^b^	0.25 ± 0.01^b^	3.1 ± 0.1	0.25 ± 0.01^b^	3.1 ± 0.1	0.27 ± 0.00^a^
	Stored (*t*_2_)	9.2 ± 0.4^b^	0.22 ± 0.02^b^	4.7 ± 0.4^a^^,^ b	0.18 ± 0.02^a^^,^ b	4.4 ± 0.5^a^	0.22 ± 0.01^b^
*E. hallii*	Fresh (*t*_0_)	1.7 ± 0.8	0.36 ± 0.04	1.5 ± 0.0	0.34 ± 0.02	1.3 ± 0.2	0.23 ± 0.02^a^
	Processed (*t*_1_)	4.1 ± 0.2	0.43 ± 0.09	3.4 ± 0.6^a^	0.41 ± 0.11	1.9 ± 0.6^a^	0.21 ± 0.04^a^
	Stored (*t*_2_)	8.1 ± 2.5^b^	0.47 ± 0.03^b^	7.3 ± 1.3^a^^,^ b	0.43 ± 0.03^a^^,^ b	5.3 ± 0.8^a^^,^ b	0.30 ± 0.02^a^^,^ b
*A. caccae*	Fresh (*t*_0_)	1.5 ± 0.1	0.20 ± 0.03	1.3 ± 0.2	0.19 ± 0.03	2.0 ± 0.3	0.21 ± 0.02
	Processed (*t*_1_)	2.4 ± 0.2^b^	0.15 ± 0.03^b^	1.8 ± 0.5^b^	0.17 ± 0.02	2.2 ± 0.1^a^	0.13 ± 0.02^b^
	Stored (*t*_2_)	3.3 ± 0.6^b^	0.16 ± 0.02	2.2 ± 0.2^a^^,^ b	0.16 ± 0.00^b^	2.1 ± 0.3^a^	0.14 ± 0.01^b^
*F. prausnitzii*	Fresh (*t*_0_)	1.3 ± 0.1	0.05 ± 0.00	1.4 ± 0.1	0.05 ± 0.00	3.6 ± 0.1^a^	0.05 ± 0.00^a^
	Processed (*t*_1_)	2.5 ± 0.2	0.05 ± 0.01	3.3 ± 0.2^a^	0.06 ± 0.00	4.5 ± 0.3^a^	0.06 ± 0.00^a^
	Stored (*t*_2_)	5.0 ± 1.1^b^	0.02 ± 0.00^b^	5.4 ± 1.4^b^	0.03 ± 0.00	5.5 ± 1.2^b^	0.03 ± 0.00^a^

a Lag phase or growth rate in samples with cryoprotectants is significantly different from the control samples within the same condition (*P* ˂ 0.05).

b Lag phase or growth rate after processing and after storage is significantly different (B) from the fresh culture within the same treatment (*P* ˂ 0.05).

### Bacterial response to cryopreservation in the absence of protectants

The effect of cryopreservation and frozen storage at −80°C for 3 months on viability and fitness of the investigated strains was evaluated by comparing processed (*t*_1_) and stored (*t*_2_) control cultures lacking protectants with the fresh control culture (*t*_0_) (Fig. 1).

*R. intestinalis*,* E. hallii* and *B. obeum* were strongly impacted by cryopreservation, indicated by significantly lower viable cell counts and increased *t*_lag_ in the processed and stored samples compared to fresh control cultures (Table 1). *R. intestinalis* and *E. hallii* exhibited a 100‐fold lower MPN after storage (7.1 ± 0.2 and 6.8 ± 0.6 log ml^−1^, respectively) compared to the fresh control (9.0 ± 0.3 and 8.4 ± 0.2 log ml^−1^, respectively), along with a fivefold to sixfold increased *t*_lag_ (Table 2). The proportion of intact *E. hallii* cells declined from 71 ± 9% in fresh to 3 ± 0% in stored control culture. *B. obeum* was the most sensitive strain towards freezing. Its MPN was strongly reduced from 7.9 ± 0.4 log ml^−1^ in fresh to 5.7 ± 0.7 log ml^−1^ after processing (Table 1), and a further decline after storage (4.5 ± 0.6 log ml^−1^). Consistently, *t*_lag_ significantly increased from 6.4 ± 0.2 h in the fresh to 19.7 ± 0.8 h in the stored *B. obeum* culture. The effect of cryopreservation on μ_max_ was species‐dependent. *B. obeum* and *E. hallii* exhibited an increase and *R. intestinalis* a decrease of μ_max_ after processing and storage (0.26 ± 0.03, 0.47 ± 0.03 and 0.22 ± 0.02 OD unit h^−1^, respectively) compared to fresh (0.14 ± 0.03, 0.36 ± 0.04 and 0.29 ± 0.02 OD unit h^−1^, respectively) (Table 2).

**Figure 2:**
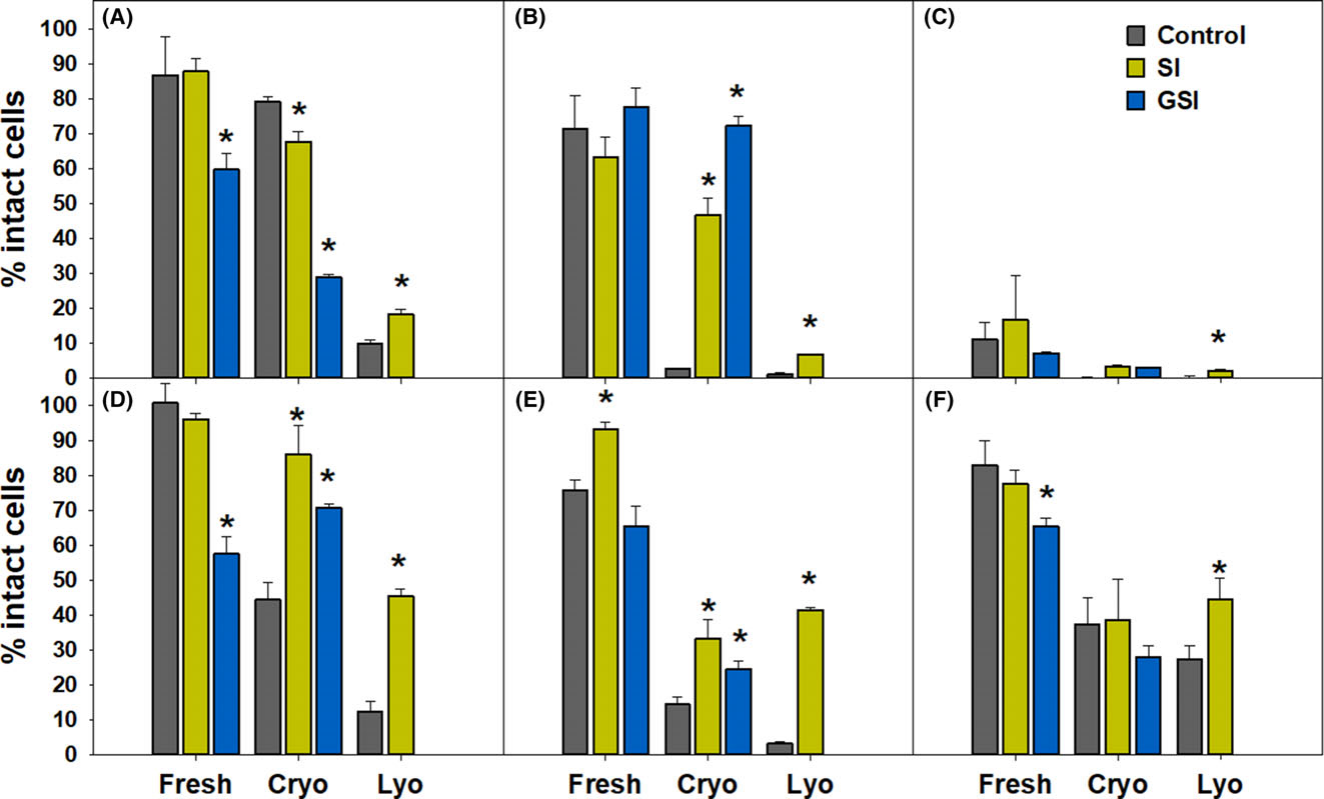
Impact of cryoprotectants on membrane integrity of cryopreserved and lyophilized strict anaerobes. Percentage of intact cells in fresh (*t*_0_), and cryopreserved (Cryo) and lyophilized (Lyo) *B. thetaiotaomicron* (A), *E. hallii* (B), *B. obeum* (C), *R. intestinalis* (D), *A. caccae* (E) and *F. prausnitzii* (F) after 3 months storage in control (no protectant) and treated cultures (*t_2_*) (SI and GSI).

*B. thetaiotaomicron*,* F. prausnitzii* and *A. caccae* were less impacted by freezing and storage as indicated by stable or little changed MPN of fresh, processed and stored cultures (Table 1). *B. thetaiotaomicron* was least sensitive, as the percentage of intact cells was not affected during storage (Fig. 2) although *t*_lag_ was slightly but significantly increased after freezing (1.1 ± 0.1 h) compared to fresh culture (0.7 ± 0.1 h). In contrast, cryopreservation and storage reduced the fraction of intact *F. prausnitzii* and *A. caccae* cells from 83 ± 7% and 63 ± 8% in the fresh to 27 ± 4% and 10 ± 3%, respectively, in the stored samples, along with a significantly increase of *t*_lag_ (Table 2). A decrease of μ_max_ from fresh to processed and stored cultures was measured for all three strains (Table 2).

### Impact of protectants on cryopreserved cultures

The protective effect of the non‐penetrating agents inulin and sucrose alone (SI) or in combination with the penetrating glycerol (GSI) on viability and fitness of cryopreserved strains was evaluated during freezing and storage at −80°C. Processed (*t*_1_) and stored (*t*_2_) SI‐ and GSI‐treated cultures were compared to fresh cultures (*t*_0_) as well as processed (*t*_1_) and stored (*t*_2_) control samples without protectants.

SI improved viability and fitness of the stored freezing‐sensitive strains, while glycerol in the protective medium (GSI) further enhanced the protective effect (Fig. S1, Tables 1 and 2). The viable cell counts of *R. intestinalis* and *B. obeum* stored in SI (7.7 ± 0.3 and 6.1 ± 0.4 log ml^−1^) and GSI (8.3 ± 0.2 and 7.0 ± 0.4 log ml^−1^) were significantly higher than without protectants (7.1 ± 0.2 and 4.5 ± 0.6 log ml^−1^). Both strains exhibited similar *t*_lag_ with SI and GSI which was significantly lower than in the stored control (Table 2). Viable cell counts and *t*_lag_ of stored *E. hallii* were also increased and decreased, respectively, with GSI (7.9 ± 0.4 log ml^−1^ and 5.3 ± 0.8 h) compared to the control (6.8 ± 0.6 log ml^−1^ and 8.1 ± 2.5 h), and no effect was shown with SI. The highest fraction of intact cells was obtained with GSI (73 ± 3%), compared to SI (47 ± 5%) and the stored control (3 ± 0%). Unexpectedly, μ_max_ of GSI‐treated *E. hallii* after storage (0.30 ± 0.02 OD unit h^−1^) was significantly lower than for the control (0.47 ± 0.03 OD unit h^−1^).

The positive impact of protectants was less distinct during processing than during storage. Only small differences in viability and *t*_lag_ of the freezing‐sensitive strains were observed between the processed control, SI‐ and GSI‐treated samples, except for *B. obeum* with higher MPN in SI (7.1 ± 0.5 log ml^−1^) and GSI (7.7 ± 0.04 log ml^−1^) than the control (5.7 ± 0.7 log ml^−1^).

Viability and fitness of *B. thetaiotaomicron*,* F. prausnitzii* and *A. caccae*, which were less sensitive towards cryopreservation, were little impacted by the addition of SI and GSI. MPNs of *B. thetaiotaomicron* and *A. caccae* did not differ between fresh, processed and stored cultures, independent from the addition of protectants. MPN of *F. prausnitzii* remained stable after cryopreservation and storage in the GSI and control samples, but were significantly lower in SI‐treated culture after storage (7.6 ± 0.3 log ml^−1^) compared to the fresh SI culture (8.3 ± 0.3 log ml^−1^).

### Bacterial response to lyophilization in the absence of protectant

The effect of lyophilization and storage at 4°C for 3 months on viability and fitness of the tested strains was evaluated by comparing processed (*t*_1_) and stored (*t*_2_) with fresh cultures (*t*_0_) in the absence of protectant (control).

Lyophilization more severely affected viability and growth than freezing only. Viable cell counts decreased 100‐fold, and *t*_lag_ increased up to 14‐fold after processing but remained stable during storage of all strains except *F. prausnitzii* and *B. obeum* (Tables 3 and 4). *F. prausnitzii* was the least sensitive strain towards lyophilization as indicated by a recovery of 14% of initial viable cells (7.1 ± 0.4 log cells ml^−1^) and a relative high fraction of intact cells (27 ± 4%) in the lyophilized stored control (Fig. 2). In contrast, *B. obeum* was most sensitive, with a large drop of viability during processing (4.3 ± 1.2 log ml^−1^) and during storage (2.3 ± 2.1) compared to the fresh control cultures (7.9 ± 0.4 log ml^−1^). For all strains except *B. obeum*, μ_max_ decreased between 14% and 60% after lyophilization and storage. A significant increase of μ_max_ from 0.14 ± 0.02 in fresh to 0.27 ± 0.00 OD unit h^−1^ in stored culture was measured for *B. obeum*.

**Table 3:** Impact of protectants and lyophilization on viable cell counts of fresh (*t*_0_), processed (*t*_1_) and stored (*t*_2_) bacteria. Log viable cell counts ml^−1^ in the fresh culture, after lyophilization and storage for 3 months at 4°C, were assessed with the most probable number method (MPN). Recovery rate of viable cells (in %) was calculated relative to the average viable cell counts in the fresh control (vs. control *t*_0_) and to fresh treatment culture (vs. *t*_0_)

Organism	Culture condition	Control		SI	
		MPN (ml^−1^)	Recovery vs. *t*_0_	MPN (ml^−1^)	Recover vs. control *t*_0_/*t*_0_
*B. thetaiotaomicron*	Fresh (*t*_0_)	9.3 ± 0.2		9.4 ± 0.2	135
	Processed (*t*_1_)	7.0 ± 0.5	1	8.0 ± 0.7^b^	6/4
	Stored (*t*_2_)	5.4 ± 2.0^b^	0.01	7.7 ± 0.4^a^^,^ b	3/2
*B. obeum*	Fresh (*t*_0_)	7.9 ± 0.4		7.8 ± 0.4	79
	Processed (*t*_1_)	4.3 ± 1.2	0.02	6.6 ± 0.2^a^^,^ b	4/5
	Stored (*t*_2_)	2.3 ± 2.1^b^	0.0003	5.5 ± 0.3^a^^,^ b	0.3/0.4
*R. intestinalis*	Fresh (*t*_0_)	8.9 ± 0.4		8.9 ± 0.4	82
	Processed (*t*_1_)	6.3 ± 0.3^b^	0.2	7.8 ± 0.4^a^^,^ b	7/8
	Stored (*t*_2_)	6.2 ± 0.2^b^	0.2	7.6 ± 0.2^a^^,^ b	4/5
*E. hallii*	Fresh (*t*_0_)	8.4 ± 0.2		8.4 ± 0.2	100
	Processed (*t*_1_)	4.9 ± 0.4^b^	0.04	7.1 ± 0.1^a^^,^ b	6/6
	Stored (*t*_2_)	5.6 ± 0.2^b^	0.2	6.8 ± 0.2^a^^,^ b	3/3
*A. caccae*	Fresh (*t*_0_)	8.8 ± 0.3		8.9 ± 0.2	111
	Processed (*t*_1_)	6.4 ± 0.2^b^	0.4	8.8 ± 0.3^a^	87/71
	Stored (*t*_2_)	6.7 ± 0.5^b^	1	8.6 ± 0.3^a^	60/49
*F. prausnitzii*	Fresh (*t*_0_)	8.0 ± 0.2		8.2 ± 0.2	181
	Processed (*t*_1_)	6.1 ± 0.3^b^	1	7.8 ± 0.4^a^^,^ b	58/32
	Stored (*t*_2_)	7.1 ± 0.4^b^	14	7.8 ± 0.1^a^^,^ b	59/33

a Viable cell counts in samples with lyoprotectants are significantly different from the control samples within the same condition (*P* ˂ 0.05).

b Viable cell counts after processing and after storage are significantly different from the viable cell counts of the fresh culture within the same treatment (*P* ˂ 0.05).

**Table 4:** Impact of protectants and lyophilization on fitness of fresh (*t*_0_) processed (*t*_1_) and stored (*t*_1_) bacteria. Lag phase (*t*_lag_) and maximum growth rate (µ_max_) were calculated from optical density growth curves based on Baranyi's equation

Organism	Culture condition	Control		SI	
		*t*_lag_ (h)	µ_max_ (OD*h^−1^)	*t*_lag_ (h)	µ_max_ (OD*h^−1^)
*B. thetaiotaomicron*	Fresh (*t*_0_)	0.7 ± 0.1	0.23 ± 0.01	0.7 ± 0.1	0.22 ± 0.01^a^
	Processed (*t*_1_)	7.5 ± 0.9^b^	0.19 ± 0.01^b^	3.4 ± 1.2^a^^,^ b	0.18 ± 0.01
	Stored (*t*_2_)	10.1 ± 3.5^b^	0.16 ± 0.04^b^	4.1 ± 1.9^a^^,^ b	0.14 ± 0.05^a^
*B. obeum*	Fresh (*t*_0_)	6.4 ± 2.2	0.14 ± 0.02	7.0 ± 2.1	0.14 ± 0.02
	Processed (*t*_1_)	25.8 ± 0.3^b^	0.25 ± 0.00^b^	19.3 ± 2.1^a^^,^ b	0.21 ± 0.01^a^^,^ b
	Stored (*t*_2_)	25.5 ± 1.6^b^	0.27 ± 0.00^b^	21.5 ± 2.0^a^^,^ b	0.22 ± 0.03^a^^,^ b
*R. intestinalis*	Fresh (*t*_0_)	1.6 ± 0.1	0.29 ± 0.02	1.6 ± 0.1	0.28 ± 0.01
	Processed (*t*_1_)	9.8 ± 0.4^b^	0.30 ± 0.01	5.6 ± 1.1^a^	0.26 ± 0.01^a^
	Stored (*t*_2_)	11.0 ± 0.5^b^	0.25 ± 0.01^b^	8.5 ± 0.4^a^^,^ b	0.21 ± 0.01^a^^,^ b
*E. hallii*	Fresh (*t*_0_)	1.7 ± 0.7	0.35 ± 0.04	1.3 ± 0.2	0.30 ± 0.07
	Processed (*t*_1_)	14.8 ± 0.2^b^	0.27 ± 0.05^b^	8.5 ± 0.7^a^^,^ b	0.37 ± 0.02^a^
	Stored (*t*_2_)	13.0 ± 1.3^b^	0.21 ± 0.12^b^	8.6 ± 0.2^a^^,^ b	0.32 ± 0.04
*A. caccae*	Fresh (*t*_0_)	1.5 ± 0.1	0.20 ± 0.03	1.3 ± 0.2	0.19 ± 0.03
	Processed (*t*_1_)	8.9 ± 0.9^b^	0.25 ± 0.02	2.7 ± 0.1^a^^,^ b	0.16 ± 0.01^a^^,^ b
	Stored (*t*_2_)	7.9 ± 0.3^b^	0.21 ± 0.01	2.6 ± 0.4^a^^,^ b	0.15 ± 0.02^a^
*F. prausnitzii*	Fresh (*t*_0_)	1.3 ± 0.1	0.05 ± 0.00	1.4 ± 0.1	0.05 ± 0.00
	Processed (*t*_1_)	6.6 ± 0.4^b^	0.04 ± 0.01^b^	3.3 ± 0.4^a^^,^ b	0.06 ± 0.01^a^
	Stored (*t*_2_)	5.7 ± 2.0	0.02 ± 0.01^b^	2.8 ± 0.7^a^^,^ b	0.03 ± 0.01^a^

a Lag phase or growth rate in samples with lyoprotectants is significantly different from the control samples within the same condition (*P* ˂ 0.05).

b Lag phase or growth rate after processing and after storage is significantly different from the fresh culture within the same treatment (*P* ˂ 0.05).

### Impact of protectants on lyophilized cultures

The protective effect of SI was evaluated on viability and fitness of lyophilized strains after processing (*t*_1_) and storage at 4°C (*t*_2_), and compared to the fresh cultures (*t*_0_) as well as to processed and stored control samples with not added protectant.

The addition of SI significantly improved viability and fitness of all lyophilized strains (Fig. S1, Tables 3 and 4), with most effect on viability recorded for *A. caccae* and *F. prausnitzii*. Viable cell counts of *A. caccae* remained unchanged after processing (8.8 ± 0.3 log ml^−1^) and after 3‐month storage (8.6 ± 0.3 log ml^−1^, 34.3% intact cells) compared to the fresh SI samples (8.9 ± 0.2 log ml^−1^, 83 ± 6% intact cells). *t*_lag_ of SI‐treated *A. caccae* and *F. prausnitzii* increased approximately twofold after processing (2.7 ± 0.1 and 2.6 ± 0.4 h, respectively) but was significantly lower than for the lyophilized and stored control (8.9 ± 0.9 and 7.9 ± 0.3 h, respectively). Viable cell counts of *F. prausnitzii* slightly but significantly decreased from 8.2 ± 0.2 log cells ml^−1^ in the fresh SI samples to 7.8 ± 0.4 log cells ml^−1^ after lyophilization, but remained stable during storage. *t*_lag_ was twofold longer after storage (2.8 ± 0.7 h) than in the fresh SI samples, however, significantly shorter than for the lyophilized control (5.7 ± 2.0 h).

*B. thetaiotaomicron*,* R. intestinalis* and *E. hallii* treated with SI had similar viable cell counts after storage (7.7 ± 0.4, 7.6 ± 0.2 and 6.8 ± 0.2 log cells ml^−1^, respectively) and improved recovery rates of 3–4% viable cells compared to the control (0.01–0.2%). SI addition also significantly shortened *t*_lag_ by 33–59% after lyophilization and storage compared to the protectant‐free control (Table 4). Viability remained generally stable during storage of all lyophilized cultures with a maximal loss of 0.3 log in MPN, except for *B. obeum* that showed a 1 log decrease in MPN and an increased μ_max_ after processing and storage of SI‐treated samples.

## Discussion

A major challenge in the production and formulation of strict anaerobic probiotics of gut origin is maintaining viability and fitness during processing and storage. In this study, we assessed the impact of the two main preservation methods, cryopreservation and lyophilization, and of subsequent storage, respectively, on viability and fitness of six strict anaerobic gut microbes.

### Impact of cryopreservation, lyophilization and storage on viability

Cryopreservation was confirmed less detrimental to sensitive bacteria than lyophilization (Heylen *et al*., 2012), which combines freezing and drying steps, as indicated by higher viability and shorter *t*_lag_ of cryopreserved compared to lyophilized samples directly after processing in the absence of cryoprotectant. During freezing, bacterial cells are exposed to two main stresses. Mechanical stress due to intra‐ and extracellular ice crystal formation and increased osmotic pressure caused by solutes in the remaining unfrozen fraction can potentially lead to the disruption of bacterial membranes and ultimately to lethal damage (Malik, 1991; Meryman, 2007). During lyophilization, the removal of water by sublimation further increases osmotic pressure and can cause severe damage to membranes and surface proteins (Broeckx *et al*., 2016). However, despite lower viable cell counts in the lyophilized cultures without protectant, viability was maintained during storage at 4°C while viability of the corresponding cryopreserved cultures declined at −80°C, especially for the freezing‐sensitive *B. obeum*,* R. intestinalis* and *E. hallii*. Biochemical reactions occurring when free water is present in cells stored above −80°C can cause viable cell loss over time (Mazur, 1970, 1984). As a consequence, storage in electrical freezers will not guarantee indefinite viability of cryopreserved cells (Heylen *et al*., 2012).

Another important factor for stability of strict anaerobes is oxidative stress. The ability to tolerate oxygen differed between the tested microbes. An aerotolerance test identified *R. intestinalis*,* F. prausnitzii* and *E. hallii* as highly oxygen‐sensitive and *A. caccae* as most oxygen‐tolerant strain withstanding exposure to ambient air up to 60 min (Flint *et al*., 2007). *Faecalibacterium prausnitzii* can survive in the presence of low oxygen concentrations using an ‘extracellular electron shuttle’ over antioxidants that reduce oxygen (Khan *et al*., 2012). *Bacteroides thetaiotaomicron* also expresses defence mechanisms against oxygen by scavenging enzymes that prevent rapid formation of reactive oxygen species and facilitates recovery from oxygen exposure (Pan and Imlay, 2001; Mishra and Imlay, 2013). By adding riboflavin and cysteine HCl in the buffer formulation, we induced a reducing environment protecting bacteria from oxygen exposure during storage; nevertheless, highly sensitive strains might require complete anaerobiosis (Khan *et al*., 2014). Higher oxygen tolerance of *A. caccae, F. prausnitzii* and *B. thetaiotaomicron* may explain their enhanced stability during storage at −80°C in partly oxygen‐permeable screw‐cap polypropylene cryo tubes. Improved stability of the lyophilized compared to cryopreserved samples might also be partly due to the absence of oxygen during storage through vacuum‐sealed glass ampules. As suggested by Malik (1991), storage stability of strict anaerobes during cryopreservation can be improved using glass vials with oxygen‐impermeable butyl rubber septa.

### Impact of protectants on viability and growth of fresh cultures

Prior preservation, fresh cultures were incubated for 30 min in protective media containing 2.0 M glycerol, 150 mM sucrose and 40 mM inulin (calculated as fructose equivalents). Solutes used as protectants can cause growth inhibition due to osmotic pressure when present in growth medium at concentrations of > 1.0 M sucrose and ≥ 1.5 M glycerol (Cebrian *et al*., 2014). Membranes, destabilized after exposure to osmotic pressure, are suggested to cause cell death by phase transition of membrane phospholipids in interaction with volume changes of the cells (Mille *et al*., 2005). Glycerol in the protective buffer already decreased survival and the proportion of intact cells of fresh *B. thetaiotaomicron*,* R. intestinalis* and *F. prausnitzii* cultures after 30 min incubation. The minimal inhibitory concentration of glycerol in growth medium (1.5–2.4 M) was found to be generally lower in Gram‐negative than Gram‐positive bacteria. It was also suggested that osmotolerance might be strain specific (Saegeman *et al*., 2008; Cebrian *et al*., 2014).

The presence of glycerol in the protective solution reduced μ_max_ of *E. hallii* compared to the SI treatment and control without glycerol. These data may be explained by the ability of *E. hallii* to convert glycerol to reuterin (Engels *et al*., 2016a). Reuterin is a broad‐spectrum antimicrobial system, which, at physiological conditions, mainly consists of 3‐hydroxypropionaldehyde (3‐HPA), its hydrate and dimer, and acrolein (Engels *et al*., 2016b). Reuterin can inhibit growth of the producer strain. In the growth assessment tests, 10% (v/v) inoculation with GSI‐treated cultures transferred 20 mM glycerol to the YCFA medium. Hence, formation of reuterin could be responsible for reduced μ_max_ of *E. hallii*, which is supported by the absence of an effect on μ_max_ when inoculation with the glycerol containing culture was performed at only 1% v/v (data not shown).

Fresh cultures incubated in protective media containing sucrose (150 mM) and inulin (40 mM) were not negatively affected in terms of viability or growth. The concentration of sucrose was likely too low to induce a significant osmotic stress. Moderate osmotic stress was shown to occur at much higher concentration of sucrose (730 mM) for *Lactobacillus delbrueckii* (Meneghel *et al*., 2017). In another study, growth inhibition of *Staphylococcus aureus*,* Listeria monocytogenes*,* Cronobacter sakazakii*,* Enterococcus faecium*,* Escherichia coli* and *Salmonella Typhimurium* was observed in growth medium supplemented with sucrose concentrations ranging from 1.1 to 1.8 M (Cebrian *et al*., 2014).

### Impact of protectants on viability and growth of preserved cultures

The addition of protectants positively influenced viability and membrane integrity of the freezing‐sensitive *R. intestinalis*,* E. hallii* and *B. obeum*. It has been proposed that glycerol prevents intracellular ice crystal formation at high freezing rates when bacterial cells are immersed in liquid nitrogen (Fonseca *et al*., 2006). The protective action of glycerol during freezing and storage also outweighed its detrimental osmotic effect observed in fresh *R. intestinalis* cultures, and the potential growth inhibition of reuterin produced by *E. hallii*. Glycerol in the protective media exhibited greater protection than sucrose and inulin alone. However, the two protectants might have acted synergistically in combination with glycerol leading to better recovery of viable cells. The observed positive impact of glycerol on *R. intestinalis* on viability and growth performance is in accordance with earlier findings of its protective effect on the re‐establishment of *Roseburia sp./E. rectale* group when cryopreserved as part of a complex artificial gut microbiota (Bircher *et al*., 2017).

*B. thetaiotaomicron*,* F. prausnitzii* and *A. caccae* were little impacted by freezing and storage. Processing conditions used in this study, characterized by a high freezing rate (immersion in liquid nitrogen) and storage temperature of −80°C, contributed to the stability of these strains, as previously reported for lactic acid‐producing starter cultures (Fonseca *et al*., 2001). In agreement, only limited effects were observed on viability and the proportion of intact cells when protectants were added, and the detrimental effect of glycerol on viability of *F. prausnitzii* was still observed after freezing and storage.

The addition of SI protected cell viability during lyophilization of all strains but had little impact on storage stability. This implies that sucrose and inulin mainly protected viability during the lyophilization process. Sucrose and inulin both interact with biological membranes and stabilize during freezing and drying (Demel *et al*., 1998; Vereyken *et al*., 2003; Schwab *et al*., 2007), and in agreement, the proportion of cells with integer membranes was higher if SI was present during lyophilization compared to controls.

### Impact of preservation on bacterial fitness

Fitness is another important marker for preservation of bacteria, which can be evaluated by μ_max_ and *t*_lag_ as a measure of reproductive potential (Sandegren *et al*., 2008; Pope *et al*., 2010; Adkar *et al*., 2017). For all tested strains, *t*_lag_ negatively correlated with viable cell numbers (Fig. S1). Alteration of generation times, a known reaction to stress exposure, has been reported before for frozen and stored bacterial cells (Squires and Hartsell, 1955; Lipson, 2015; Adkar *et al*., 2017). Indeed, μ_max_ of *B. thetaiotaomicron*,* R. intestinalis*,* A. caccae* and *F. prausnitzii* generally decreased after preservation with only small differences between treatments and control. In contrast, cryopreserved *E. hallii,* and lyophilized and cryopreserved *B. obeum* exhibited increased μ_max_, particularly for the treatments characterized by low viability. Stress exposure during lyophilization and cryopreservation, which was lethal to the majority of the cells, might have selected for drying and/or freezing resistant subpopulations (Patra and Klumpp, 2013; Wang *et al*., 2014).

Enhanced stress resistance of bacteria can be an advantage for probiotic applications. In contrast, reduced bacterial fitness leading to slower growth may impair the ability of preserved bacteria to multiply and be metabolically active in the gastrointestinal tract. Still, further evaluation is needed to better understand the interplay between *t*_lag_, μ_max_ and viability in terms of probiotic application success. As an example, higher viability of cryopreserved *E. hallii* due to the presence of glycerol might compensate for the lower μ_max_. We recently reported that *E. hallii* was little impacted by freezing within a complex artificial gut microbiota, as indicated by comparable growth of fresh and preserved microbiota in a standardized growth assay (Bircher *et al*., 2017). Our current data suggest that the enhanced μ_max_ of *E. hallii* after cryopreservation could explain its competitiveness in batch fermentation. Furthermore, process‐impacted bacterial fitness can be a determinant for the selection of a preservation method that offers best conditions for probiotic re‐establishment *in vivo*. *F. prausnitzii,* for example, exhibited a shorter *t*_lag_ but similar viable cell counts after lyophilization than after cryopreservation when SI was used as protectant, suggesting lyophilization as favourable preservation method for this strain.

## Conclusion

To our best knowledge, this is the first study to date that quantitatively compared the effect of cryopreservation and lyophilization and the addition of selected protectants on viability and fitness of six strict anaerobic gut microbes. Viable cell recovery ranged from 11% to 100% after frozen and from 0.3% to 60% after dried storage, pointing towards a strong species‐dependent resistance to freezing and freeze‐drying. Membrane composition might be a determining factor as the addition of membrane‐interacting protectants sucrose and inulin improved viability of all lyophilized strain and of freezing‐sensitive strains after cryopreservation. As glycerol also differently affected strain viability and membrane integrity prior and postcryopreservation, our results suggest that selection of protectants has to be process‐ and species‐specific. Based on our results, we recommend using cryopreservation with GSI for *B. obeum*,* R. intestinalis*,* E. hallii* and *A. caccae* and SI for *B. thetaiotaomicron*. *F. prausnitzii* should be preferably preserved by lyophilization with SI.

## Experimental procedures

### Bacterial strains and culture conditions

*B. thetaiotaomicron* DSM 2079, *B. obeum* DSM 25238, *R. intestinalis* DSM 14610, *E. hallii* DSM 3353, *A. caccae* DSM 14662 and *F. prausnitzii* DSM 17677 were obtained from the Deutsche Sammlung für Mikroorganismen und Zellkulturen (Braunschweig, Germany). Bacterial pellets from 1 ml overnight growing cultures were snap‐frozen in 100 μl phosphate buffer (pH 6.8, 0.1 M) (PB, Table S1) supplemented with glycerol (15% v/w) (VWR International AG, Dietikon, Switzerland) and stored at −80°C (stock cultures). For each experiment, a fresh stock culture was thawed in an anaerobic chamber (10% CO_2_, 5% H_2_ and 85% N_2_) (Coy Laboratories, Grass Lake, Michigan, USA) and re‐suspended with 900 μl phosphate‐buffered saline (0.8% v/w, pH 6.8) (PBS, Table S2) to a final volume of 1 ml. Half a millilitre of reactivated culture was transferred to 10 ml yeast extract, casitone and fatty acid medium (YCFA) in a Hungate tube and incubated anaerobically for 10 h at 37°C to obtain a working culture. YCFA medium was prepared as described previously (Duncan *et al*., 2003) with slight modifications. Glucose (6 g l^−1^, Sigma‐Aldrich, Buchs, Switzerland) was added as sole carbon source. All components except cysteine HCl (0.01% v/w, Sigma‐Aldrich) were dissolved in deionized water, and pH was adjusted to 7.4 with 2.5 N NaOH to obtain a pH of 6.8 after autoclaving. The medium was boiled while flushing with CO_2_ until a colour change from blue to pink occured, caused by the addition of the indicator resazurin (0.1% of 1 mg ml^−1^ stock solution). Cysteine HCl was then added, and the medium was dispensed in Hungate tubes flushed with CO_2_ before autoclaving.

### Preparation of protective buffers

All components of the PB (0.1 M, prepared in oxygen‐free distilled water) were placed in an anaerobic chamber overnight to remove traces of oxygen. Reducing agents cysteine HCl and riboflavin (Sigma‐Aldrich) were added at final concentrations of 1 g l^−1^ and 0.3 g l^−1^, respectively, to protect the bacteria from potential oxygen exposure during processing and storage (Khan *et al*., 2014). The pH was adjusted to 6.8 and buffers were filter‐sterilized, covered in aluminium foil as light protection and stored in an anaerobic chamber until usage.

Two protective buffers were prepared by dissolving in PB sucrose (VWR International AG) and inulin (RPN Foodtechnology AG, Sursee, Switzerland) (both 5% w/v) (SI) and adding glycerol (15% v/w) to SI (GSI). PB that only contained cysteine HCl and riboflavin served as protectant‐free control.

### Cryopreservation, lyophilization and storage

Two independent preservation experiments were conducted for each bacterium. Within an experiment, fresh, processed and stored samples of each treatment were analysed in triplicates. All processing steps were either executed in an anaerobic chamber or in Hungate tubes to guarantee anoxic conditions. A working culture was generated by incubating a reactivated glycerol stock culture for 10 h at 37°C in YCFA medium. This working culture was once subcultured under the same conditions to obtain a viable and active culture for preservation trials (experimental culture). For the production of experimental cultures, 10 ml YCFA medium were inoculated at 0.2% (v/v) with a working culture of *B. thetaiotaomicron*,* R. intestinalis*,* E. hallii* and *A. caccae* or at 2% (v/v) for the slow‐growing strains *F. prausnitzii* and *B. obeum*. Cultures were incubated at 37°C for 13 to 15 h, depending on the strain, until early stationary growth phase was reached. Incubation times were assessed in preliminary growth tests in Hungate tubes at 37°C. Optical density (OD) at 600 nm was monitored during incubation (data not shown). Cells were harvested by centrifugation at 4°C for 10 min at 3000 *g*. The pellet was washed in 5 ml PB, centrifuged and re‐suspended in either 1 ml control, SI or GSI buffer (10‐fold concentration of the initial experimental culture). After an incubation time of 30 min at room temperature to allow penetration of glycerol, aliquots (100 μl) were snap‐frozen in liquid nitrogen and either stored at −80°C in screw‐cap polypropylene cryotubes (Bioswisstec AG, Schaffhausen, Switzerland) (control, SI and GSI cultures, Fig. 1) or dried with a manifold freeze‐dryer (VirTis BenchTop 2K, MultiTemp Scientific AG, Kloten, Switzerland). Lyophilization of the control and SI‐treated cultures was carried out in long‐stem Vacule cryogenic ampules (Sigma‐Aldrich) that were prereduced in an anaerobic chamber, plugged with sterile cotton wool, and contained blue silica gel with moisture indicator (Sigma‐Aldrich). Frozen samples in cryogenic ampules were placed on dry‐ice prior lyophilization to prevent thawing of the culture until vacuum was started. Freeze‐drying was conducted at a condenser temperature of −80°C at 80 mTorr for 6 h after which ampules were flame sealed under vacuum and stored at 4°C.

Viability assessment and growth tests were conducted under anaerobic conditions at three different time points (Fig. 1): first with the fresh culture after incubation in control and protective buffers (*t*_0_), then with the processed culture immediately after freezing, respectively, lyophilization (*t*_1_), and finally with the stored culture after 3 months at −80°C and 4°C for cryopreservation and lyophilization, respectively (*t*_2_). Each experimental condition was duplicated, and triplicate samples of each treatment were analysed. Prior to the viability and growth tests, fresh cultures (*t*_0_) were re‐suspended in 900 μl PBS immediately after incubation in the buffers. Cryopreserved cultures (*t*_1_ and *t*_2_) were transferred from −80°C freezer to an anaerobic chamber and thawed at room temperature before re‐suspending in 900 μl PBS buffer. Lyophilized cultures (*t*_1_ and *t*_2_) were rehydrated in 1 ml PBS for 1 h in an anaerobic chamber.

### Measurement of viability

Viable cell counts of were assessed by the most probable number (MPN) method with a five‐replicate design (Sutton, 2010) that was adapted to 96‐well microtiter plates (Kuai *et al*., 2001). Before use, plates (Bioswisstec AG) were stored overnight in the anaerobic chamber to remove traces of oxygen. Samples were serially diluted tenfold, and 20 μl of each dilution was used to inoculate 5 wells, each containing 180 μl YCFA medium. Plates were incubated at 37°C for 48 h in an anaerobic chamber. Wells with visible turbidity were scored as growth positive.

### Determination of membrane integrity

Membrane integrity of fresh and stored samples was determined with two fluorescence stains and subsequent flow cytometric analysis (Van Nevel *et al*., 2013). All dilution, staining and incubation steps were performed in an anaerobic chamber while flow cytometric analysis was conducted at ambient air. Fluorescence working solutions were prepared as follows: 10 μl SYBR Green I (SG; 10 000× concentrated) (Life Technologies, Zug, Switzerland) was diluted in 990 μl filtered dimethyl sulphoxide (DMSO) (Sigma‐Aldrich). Twenty microlitre propidium iodide (PI; 20 mM) (Life Technologies) and 10 μl SG were diluted in 970 μl DMSO. Solutions were stored at −20°C until use. Each bacterial sample was diluted to approximately 10^7^ cells ml^−1^ with PBS and stained twice with SG to assess total cell counts, or with SG combined with PI to determine intact cell counts. Therefore, 30 μl diluted sample, 3 μl stain working solution and 237 μl PBS were incubated for 22 min at 37°C in the dark. PBS stained with SG and PI was used to determine background fluorescence. Prior to flow cytometric analysis, 30 μl of Flow‐Count fluorospheres (Beckman Coulter International SA, Nyon, Switzerland) was added at known concentrations to determine bacterial cell counts. Samples were analysed with a Cytomics FC 500 (Beckman Coulter International SA) equipped with an air‐cooled argon ion laser emitting 20 mW at 488 nm and a red solid state diode laser emitting 25 mW at 633 nm. Gates on green against red fluorescence plots were used to assess total and intact cell counts. The percentage of intact cells were calculated by dividing the intact cell number in the green gate of the SG and PI by the total cell number in the SG‐stained sample.

### Assessment of growth performance

Growth was assessed in 96‐well microtiter plates in anaerobic conditions, as described previously (Eini *et al*., 2013; Geirnaert *et al*., 2014). Inner wells of a 96‐well plate, stored overnight in an anaerobic chamber, were filled with 180 μl YCFA medium. Wells were inoculated with 20 μl (10% v/v) of the tested culture samples. Empty wells in outer rows and columns were filled with the reducing agent of an AnaeroGen bag (Thermo Fisher Diagnostics AG, Pratteln, Switzerland) to maintain an oxygen‐free atmosphere when plates were moved outside of the anaerobic chamber. Plates were covered with a ClearSeal film (Labgene Scientific Instruments, Châtel‐Saint‐Denis, Switzerland) and the lid, which was sealed with petroleum jelly. Growth was monitored at 37°C by measuring OD at 600 nm every 30 minutes in a microplate reader (Powerwave XS). The addition of the indicator resazurin in the YCFA medium confirmed anaerobiosis in the 96‐well plates during measurements. The average OD value of six wells containing sterile YCFA medium served as blank and was subtracted from OD values of inoculated wells. To calculate maximum growth rate (μ_max_) and lag phase (*t*_lag_), growth curves were fitted using the DMFit 3.5 program (Institute of Food Research, Norwich, UK) based on Baranyi's equation (Baranyi and Roberts, 1994).

### Statistics

Statistical analysis of viable cell counts (log_10_‐transformed), percentage of intact cells by flow cytometry, μ_max_ and *t*_lag_ were performed using R studio version 3.4.1 (Boston, Massachusetts, USA). Data are expressed as mean ± SD of six replicates obtained from two independent preservation experiments conducted on two different days (with three replicates each).

ANOVA tests were performed with viable cell counts, μ_max_ and *t*_lag_ of fresh, cryopreserved and lyophilized samples as dependant variables and either treatment or time point within a treatment as independent variables. Data were tested for normal distribution using the Shapiro–Wilk test, and equality of variance was assessed with the Levene test. Tukey HSD test (multiple pairwise comparison) was used to compare treatments to control (no added protectants) and preserved and stored to fresh samples. A nonparametric Kruskal–Wallis test was performed when data were not normally distributed or the assumption of equality of variance was violated. Student's *t*‐test was performed to compare means of viable cell counts, μ_max_ and *t*_lag_ of fresh and lyophilized control with SI‐treated samples. Data were tested for homogeneity of variance with the *F*‐test. Differences were considered significant for α ≤ 0.05.

## Conflict of interest

The authors declare no conflict of interest.

## Supporting information


**Fig. S1.** Correlation plots between viable cell counts and lag times (*t*_lag_).
**Table S1.** Composition of phosphate buffer.
**Table S2.** Composition of phosphate buffered saline.
**Table S3.** Composition of YCFA medium.
