# Supplementary material for: Effect of cryopreservation and lyophilization on viability and growth of strict anaerobic human gut microbes
Source: Microb Biotechnol. 2018 Apr 17;11(4):721–33. doi: 10.1111/1751-7915.13265 (PMC6011992; doi:10.1111/1751-7915.13265)
Supplement: Supplementary file 1 — Fig. S1. Correlation plots between viable cell counts and lag times (t lag). Table S1. Composition of phosphate buffer. Table S2. Composition of phosphate buffered saline. Table S3. Composition of YCFA medium. [file MBT2-11-721-s001.docx]

**
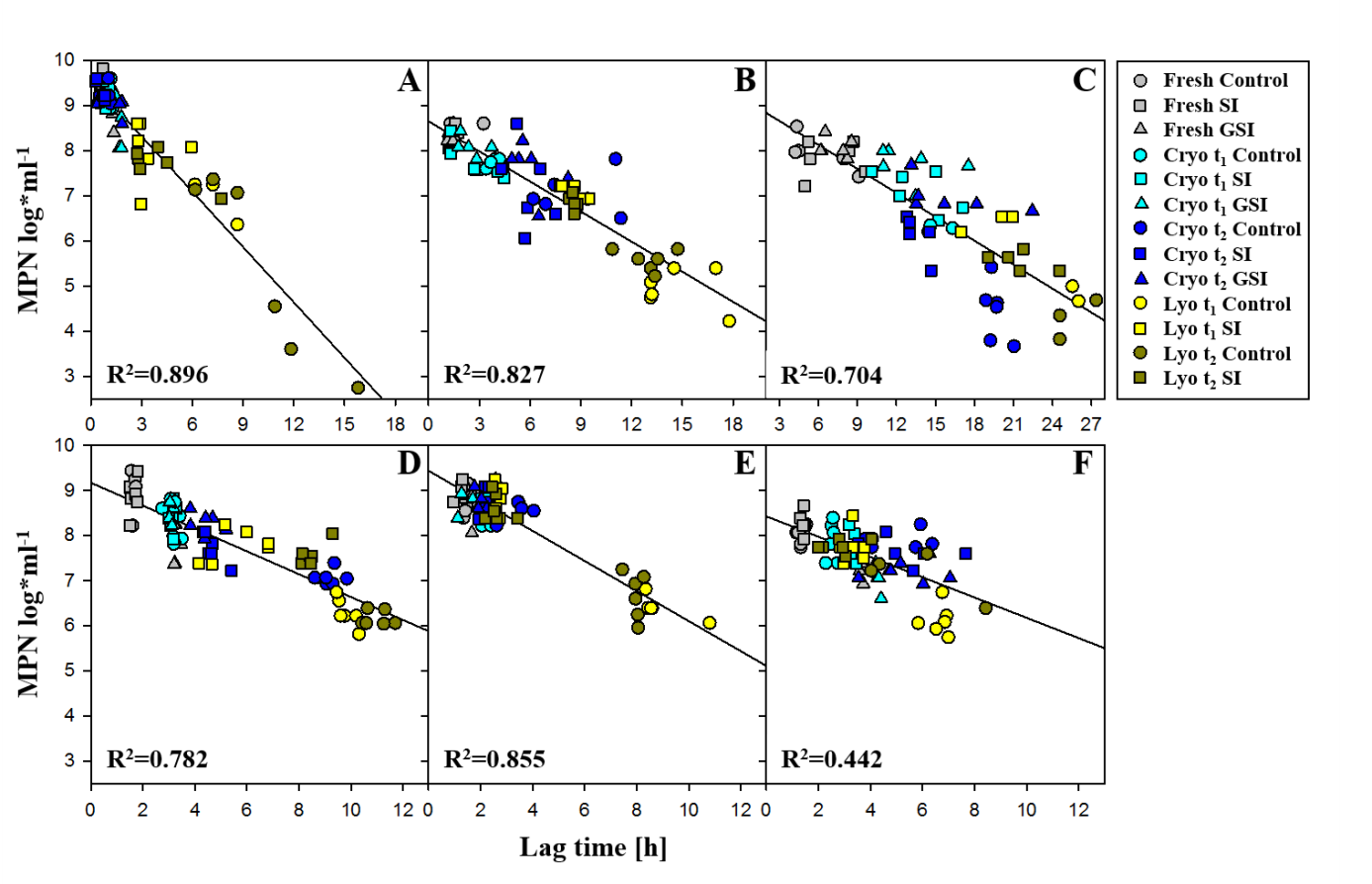
**

**Figure S.1 Correlation plots between viable cell counts and lag times (t_lag_).** Log viable cell counts and t_lag_ of fresh, preserved and stored B. thetaiotaomicron (A), E. hallii (B), B. obeum (C), R. intestinalis (D), A. caccae (E) and F. prausnitzii (F), assessed with the most probable number method (MPN) and optical density measurements, respectively.

**Table S.1** Composition of phosphate buffer.

| **Component** | **g L^-1^** |
| --- | --- |
| Sodium dihydrogen phosphate | 6.00 |
| Sodium hydrogen phosphate | 7.10 |
| Resazurin (1mg ml^-1^) | 1.00 ml |
| L-cysteine hydrochloride monohydrate | 1.00 |

**Table S.2** Composition of phosphate buffered saline.

| **Component** | **g L^-1^** |
| --- | --- |
| Potassium dihydrogen phosphate | 0.24 |
| Sodium hydrogen phosphate | 1.44 |
| Sodium chloride | 8.00 |
| Potassium cloride | 0.20 |
| Resazurin (1mg ml^-1^) | 1.00 ml |
| L-cysteine hydrochloride monohydrate | 1.00 |

**Table S.3** Composition of YCFA medium.

| **Component** | **g L^-1^** |
| --- | --- |
| Casein acid hydrolysate, from bovine milk | 10.00 |
| Yeast extract | 2.50 |
| Sodium bicarbonate | 4.00 |
| Glucose | 6.00 |
| Potassium dihydrogen phosphate  Dipotassium hydrogen phosphate  Sodium chloride  Ammonium sulfate  Magnesium sulfate  Calcium chloride | 0.45  0.45  0.90  0.90  0.09  0.09 |
| Vitamin solution (10 mg l^-1^ biotin, 10 mg l^-1^ cobalamin, 30 mg l^-1^ p-aminobenzoic acid, 50 mg l^-1^ folic acid, 150 mg l^-1^ pyridoxamine) | 1.00 ml |
| Volatile fatty acid mix (32.9% (v/v) acetic acid, 11.7% (v/v) propionic acid, 1.6% (v/v) isobutyric acid, 1.9% (v/v) isovaleric acid, 1.9% (v/v) valeric acid, 50% (v/v) 2.5 M NaOH) | 5.75 ml |
| Hemin (0.5 mg ml^-1^) | 0.20 ml |
| Resazurin (1 mg ml^-1^) | 1.00 ml |
| L-cysteine hydrochloride monohydrate | 1.00 |
